# Comparison of the effects of negative pressure wound therapy and negative pressure wound therapy with instillation on wound healing in a porcine model

**DOI:** 10.3389/fsurg.2023.1080838

**Published:** 2023-04-17

**Authors:** Sun Tingting, Feng Xinyue, Yang Tiantian, An xiao, Li Rui, Lin Feng, Liu Daohong, Li Zhirui, Wang Guoqi

**Affiliations:** ^1^ Department of Orthopaedics, Hainan Hospital of Chinese PLA General Hospital, Sanya, China; ^2^ Department of Dermatology, Hainan Hospital of Chinese PLA General Hospital, Sanya, China; ^3^ Department of Anesthesiology, Wenchang People’s Hospital, Wenchang, China; ^4^ Senior Department of Orthopedics, The Fourth Medical Center of PLA General Hospital, Beijing, China; ^5^ Department of Orthopedics, The Eighth Medical Center of PLA General Hospital, Beijing, China; ^6^ National Clinical Research Center for Orthopedics, Sports Medicine & Rehabilitation, PLA General Hospital, Beijing, China; ^7^ Department of Pediatric, The First Medical Center of Chinese PLA General Hospital, Beijing, China; ^8^ Senior Department of Pediatric, The Seventh Medical Center of Chinese PLA General Hospital, Beijing, China

**Keywords:** NPWT, instillation, wound healing, staphylococcus aureus, virulence

## Abstract

**Background:**

Negative pressure wound therapy with instillation (NPWTi) is a novel method based on standard negative pressure wound therapy (NPWT). This study aimed to compare the effects of standard NPWT and NPWTi on bioburden and wound healing in a *Staphylococcus aureus* (*S.aureus*) infected porcine model.

**Methods:**

Green fluorescent protein-labeled *S.aureus* infected wounds were created on the back of porcine. Wounds were treated with NPWT or NPWT with instillation (saline). The tissue specimens were harvested on days 0 (12 h after bacterial inoculation), 2, 4, 6, and 8 at the center of wound beds. Viable bacterial counts, laser scanning confocal microscopy, PCR, western blot, and histological analysis were performed to assess virulence and wound healing.

**Results:**

The bacterial count in the NPWTi group was lower than that of the NPWT group and the difference was statistically significant on day 2, day 4, day 6, and day 8 (*P* < 0.05). The expression levels of agrA, *Eap*, *Spa,* and *Hla* genes of the NPWTi group were significantly lower than that of the NPWT group on day 8 (*P* < 0.05). The bacterial invasion depth of the NPWTi group was significantly lower than that of the NPWT group on day 2, day 4, day 6, and day 8 (*P* < 0.05). Though the NPWTi group showed a significantly increased expression of *bFGF* and *VEGF* than that of the NPWT group in the early time (*P* < 0.05), NPWTi cannot lead to better histologic parameters than the NPWT group (*P* > 0.05).

**Conclusion:**

Our results demonstrated that NPWTi induced a better decrease in bacterial burden and virulence compared with standard NPWT. These advantages did not result in better histologic parameters on the porcine wound model.

## Introduction

Treatment of infected wounds with soft tissue defects was very tricky. A heavy bacterial bioburden in the wound can negatively impact wound healing by stimulating a pro-inflammatory environment and encouraging the in-migration of monocytes, macrophages, and leucocytes (1). In addition, bacterial bioburden can also increase metabolic requirements and decrease blood flow to the wound, which delays wound healing (2, 3). Thus, effective control and clearance of infections are essential for normal wound healing. *Staphylococcus aureus* (*S.aureus*) is the most commonly gram-positive bacteria in infected wounds (4). *S.aureus* can express various virulence factors, such as extracellular adherence protein (Eap), staphylococcal protein A (Spa), and α-hemolysin (Hla) which play an important role in the pathogenesis of wound infection (5, 6). In addition, accessory gene regulator system (agr), a global regulatory system of *S.aureus*, plays an important role in the transcription and expression of virulence factors.

Negative pressure wound therapy (NPWT) is widely used for the management of both acute and chronic wounds (7). The benefit of NPWT has been proposed: fluid removal, macro-deformation or wound shrinkage, micro deformation at the foam-wound surface interface, and stabilization of the wound environment (8). In addition, other studies suggest that NPWT decreases edema, decreases bacterial bioburden, increases immune response, increases blood flow to the wound margins, and decreases the permeability of vessels (9–12). It has been reported that negative pressure can inhibit the growth, virulence, and biofilm formation of *S.aureus* and *P.aeruginosa* (13–15).

Negative pressure wound therapy with instillation (NPWTi) is a novel method based on standard negative pressure wound therapy, which combines NPWT and the timed delivery of topical irrigation solutions (saline, distilled water, or antimicrobials) to the wound bed (16). This method subjects the subcutaneous layer of the wound to irrigation and negative pressure, thus removing any exudates and irritants that can accumulate in the wound (17). Wolvos et al. showed that NPWTi may help with noncontaminated wound management (18). However, the possible mechanism of NPWTi on bacterial bioburden in soft tissue is unclear. Thus, we designed an animal study aimed at the comparison of the effects of NPWT and NPWTi in a porcine model of bacterial bioburden. We hypothesized that NPWTi would have beneficial effects on decreasing the number of bacteria, reducing the virulence of bacteria and the depth of bacterial invasion, which may help with wound healing.

## Materials and methods

*Ethical statement*. All animal experiments in this study were approved by the Medical Ethics Committee of the Chinese PLA General Hospital (Beijing, China) and received humane care in compliance with the Guidelines for Care and Use of Animals in Research.

*Animals.* Ten healthy pigs of both sexes, with a mean body weight of 15 kg, were used for this study. All to the animals were free to access water in separate cages under constant temperature (22°C) and humidity (45%).

*Bacterial strains and culture. S. aureus* strain RN6390-GFP (constitutively expressing a green fluorescent protein, a gift from the People's Liberation Army Institute for Disease Control and Control) was utilized for wound inoculation. *S. aureus* was cultured in Luria broth at 37°C and grown overnight until the log phase. Bacteria were harvested and diluted with phosphate-buffered saline (PBS) until OD (optical density) 600 nm reached a value of 1.0, equivalent to 10^5^ colony-forming units/µl empirically.

*Wound protocol and bacterial inoculation.* Pigs were anesthetized by intramuscular injection of ketamine (30 mg/kg) and midazolam (500 µg/kg). The dorsum of the animal was cleaned, treated with depilatory cream (Veet) for hair removal, sterilized twice with povidone and 70% ethanol, and covered with sterile surgical drapes. Two circular full-thickness 5 cm diameter excisional wounds to the muscle were created on the dorsum, with the epidermal, dermal, subdermal fat, and subcutaneous fat layers removed. Light pressure with sterile gauze was applied to stanch bleeding if necessary. Each wound was inoculated with 10^5^ colony-forming units/µl *S.aureus* at a volume of 1 ml. Bacteria were allowed to proliferate for 12 h to ensure bacterial adhesion and colonization.

*Treatment protocol.* Overall, 20 acute *S.aureus*-contaminated wounds were created (2 wounds/pig). Five pigs were randomly assigned to the NPWT (Shandong, WEIGAO) group and the others were assigned to the NPWTi group. The wound was filled with black polyurethane foam. In the NPWT group, the foam was connected to a therapy unit set to deliver intermittent negative pressure, with each cycle consisting of 5 min at -125 mmHg followed by 2 min at 0 mmHg (19). In the NPWTi group, the treatment is programmed to cycle instill the saline for 45 s, follow by a 3 second hold time to allow the solution distribution over the wound bed, then deliver 2 h of negative pressure therapy same as the NPWT group (16). Animals were anesthetized before dressing change. Foams were checked daily and changed on postoperative days 2, 4, 6, and 8. Before dressing changes, the diameter of the wounds were measured and 5 mm punch biopsies were taken from the wound for analysis and detection. The detection was repeated 3 times. Animals were sacrificed on postoperative day 8 *via* over-dose of intravenous ketamine and midazolam.

*Bacterial count.* The tissue specimens were collected 12 h after bacterial inoculation and on postoperative days 2, 4, 6, and 8. Each specimen was weighed and homogenized under sterile conditions. The homogenates were serially diluted with sterile PBS, the concentration ranging from 10^−1^, 10^−2^ to 10^−6^ times, then plated on *Staphylococcus* Isolation Ager and incubated at 37°C for 24 h. A standard colony-counting method was conducted and the results were evaluated by a medical microbiologist. The colony-counting method was conducted and the results were expressed as the logarithm of CFUs/wound (20).

*Quantitative RT-PCR.* The primer sequences used in this study were listed in Table 1. Total RNA was extracted using an RNA prep Pure Kit (TIANGEN, China) according to the manufacturer's instructions. Total RNA was treated with Recombinant DNase I (TAKARA, Japan) and reverse-transcribed using the TIANScript RT Kit (TIANGEN) according to the manufacturer's instructions. Real-time PCR analyses using the SYBR FAST qPCR Kit Master Mix Universal (KAPA, United States) were performed with an ABI7900HT sequence detection system (ABI, United States). The reaction procedures were as follows: incubation at 95°C for 3 min, and then 40 cycles at 95°C for 3 s, 60°C for the 20 s, and one dissociation step at 95°C for 15 s, 60°C for 15 s, and 95 for 15 s. All samples were analyzed in triplicate and normalized against housekeeping gene β-actin expression.

**Table 1:** Primer sequences for quantitative RT-PCR.

Gene	Primer
*Eap*	Forward: CTGTGGCAGGTAATGAGGTATCT
	Reverse: TTGAAAGCCATAATGCCATCTAC
*Spa*	Forward: GCACTACTGCTGACAAAATTGCT
	Reverse: TCCACCAAATACAGTTGTACCGA
*Hla*	Forward: TCTCTATTATCTTCAGGGTTTTCAC
	Reverse: CGAACTCGTTCGTATATTACATCTA
*agrA*	Forward: CTGATAATTCCTTATGAGGTGCTTG
	Reverse: TGCTTACGAATTTCACTGCCTA
***16S***	Forward: AGTGAAAGACGGTCTTGCTGTC
	Reverse: ATTGCGGAAGATTCCCTACTG
*VEGF*	Forward: TATGCGGATCAAACCTCACC
	Reverse: CTTGCCTCGCTCTATCTTTCTT
*bFGF*	Forward: AGTTGGTATGTGGCACTGAAA
	Reverse: GCTCTTAGCAGACATTGGAAGA
**actin**	Forward: TGGGACGACATGGAGAAGAT
	Reverse: TCTTCTCACGGTTGGCTTTG

*Western blot analysis.* The tissue specimens harvested above were homogenized and incubated on ice for 30 min in the presence of RadioImmuno Precipitation Assay (RIPA) buffer. Supernatants were collected by centrifugation at 13,000 rpm for 30 min (4°C). 25 ul of supernatant was loaded on 8% SDS-PAGE gels. Western Blot analysis for the detection of Eap, Spa, and a-toxin in wound extracts was performed as previously described (21). Antibodies for the detection of Eap, Spa, and a-toxin were purchased from Abcam.

**Figure 1:**
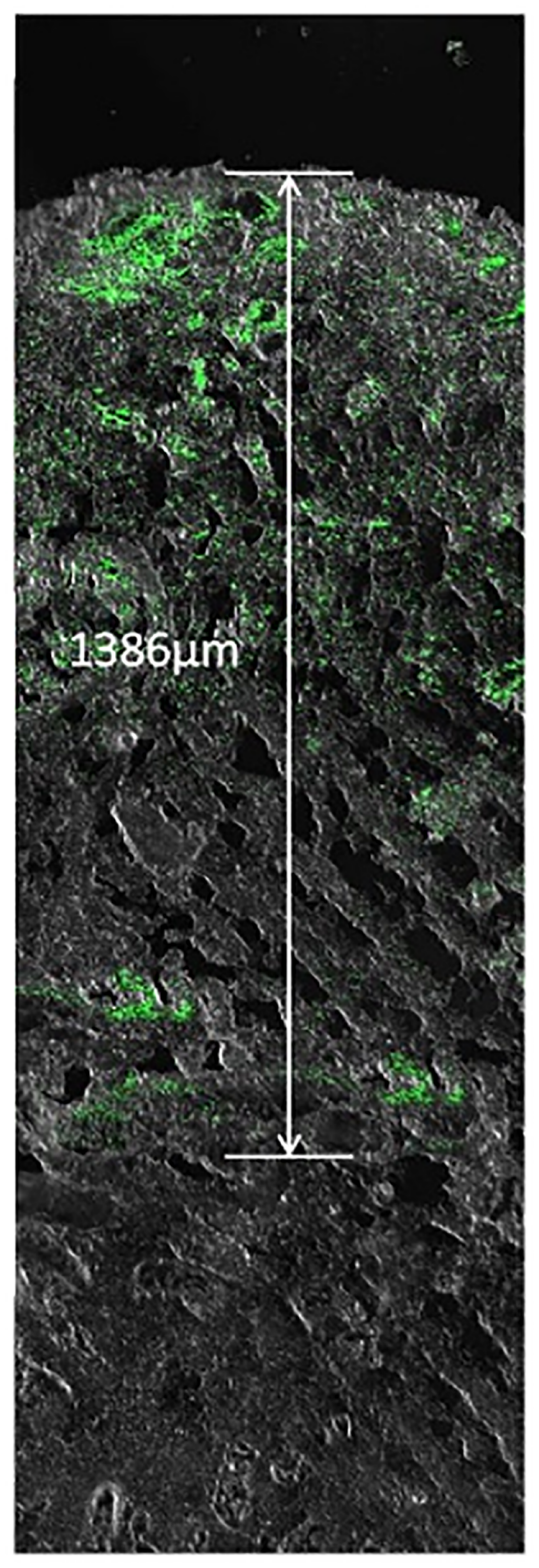
The depths of the bacterial fluorescent signal from samples.

*Measurement of bacterial invasion depth.* The muscle specimens were cut into 6 μm thick sections with the use of a cryostat and mounted on glass slides for viewing using an argon confocal laser scanning microscope (Olympus FV1000, Tokyo, Japan). *S. aureus* (constitutively expressing green fluorescent protein) was green under Olympus FV1000. To determine the invasion depth of *S.aureus* from the tissue boundary to the deepest location, digital images were captured with the speed set at 4 ms/pixel and measured by two blinded independent observers. The depths of the bacterial fluorescent signal from samples quantified by FV10-ASW 4.1 software embedded on Olympus FV-1,000 (Figure 1).

*Histological analysis.* Specimens were fixed in 10% neutral formalin. Samples were then embedded in paraffin, cut into 5 um sections, and stained for analysis. Hematoxylin and eosin (H & E) staining, immunohistochemistry (CD31), and Masson staining were performed respectively for further analysis. Image-Pro Plus version 6.0 software (Media Cybernetics, United States) was used to quantify the area of neovascularization and new granulation thickness.

*Statistical analysis.* Time course measurements were analyzed by two-way analysis of variance with repeated measures. To calculate significant differences at each time point between the two groups, a Student's *t*-test was applied. The statistical analysis was performed using SPSS software. Significant differences were defined at *P* < 0.05.

## Results

### Bacterial count

**Figure 2:**
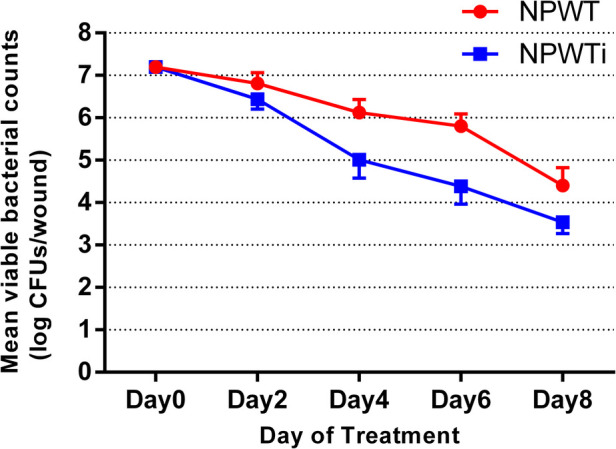
Difference in bacterial count in the two treatment groups over time (*N* = 3).

Before treatment, the bacterial count was 10^7^ CFUs of g tissue in both groups (Figure 2). In the two groups, the count of bacteria decreased from postoperative day 2, and there was a significant difference between the two groups. In the NPWTi group, the bacterial count was 10^5^ CFUs of g tissue on postoperative day 4, and less than 10^5^ CFUs of g tissue on postoperative days 6 and 8. In the NPWT group, the bacterial count was less than 10^5^ CFUs of g tissue only on postoperative day 8. Applying a two-sided *t*-test, the numbers of bacteria in the NPWT group compared with the NPWTi group was significantly different on a postoperative day 2, 4, 6, 8 (1.29 ± 0.12 × 10^7^ vs. 7.26 ± 0.13 × 10^6^, 1.02 ± 0.1 × 10^5^ vs. 7.11 ± 1.58 × 10^4^, 9.53 ± 0.62 × 10^5^ vs. 2.30 ± 0.74 × 10^4^, 2.58 ± 0.37 × 10^4^ vs. 2.78 ± 0.49 × 10^3^, all *P* < 0.05).

### Virulence

#### Virulence factor regulated gene and *agrA*

**Figure 3:**
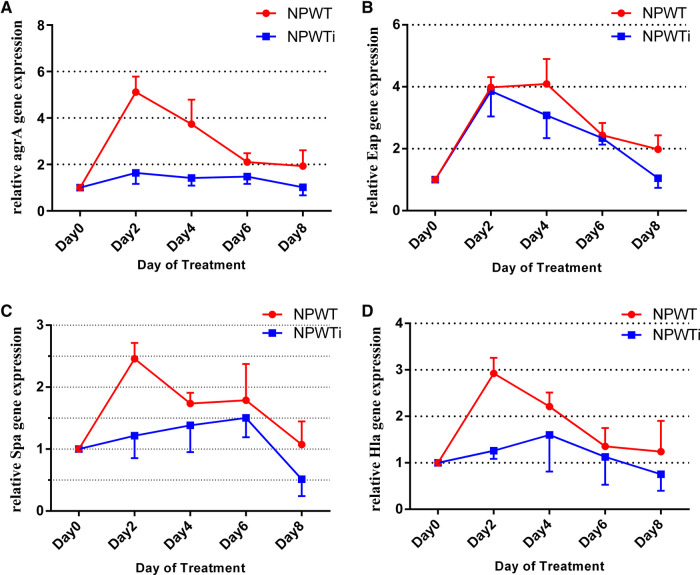
Differences in gene expression in the two treatment groups over time (fold change). (**A**) The difference of *agrA* expression; (**B**) The difference of *Eap* expression; (**C**) The difference of *Spa* expression; (**D**) The difference of *Hla* expression.

Biopsies taken from postoperative days 2, 4, 6, and 8 were analyzed by quantitative RT-PCR. Expression levels of *Eap, Spa, Hla,* and *agrA* genes were measured. Data show no statistical difference before treatment in the two groups. Data showed a trend for a significant difference in expressions of *Eap* between NPWT and NPWTi groups on a postoperative day 4 and 8 (4.10 ± 0.29 vs. 3.09 ± 0.33, 1.99 ± 0.17 vs. 1.05 ± 0.15, all *P* < 0.05), despite no statistical difference on postoperative day 2 and 6 (3.98 ± 0.13 vs. 3.87 ± 0.38, 2.44 ± 0.14 vs. 2.34 ± 0.09, all *P* > 0.05) (Figure 3). In two groups, expression levels of *Eap, Spa, Hla,* and *agrA* on postoperative days 2, 4, and 6, were higher than before treatment (Figure 3). On postoperative day 8, expression levels of *Spa* were lower than before treatment in the NPWTi group but higher in the NPWT group. The expression levels of *Spa, Hla*, and *agrA* were significantly different on a postoperative day 2, 4, 6, and 8 between NPWT and NPWTi groups (*Spa*: 2.43 ± 0.10 vs. 1.22 ± 0.17, 1.71 ± 0.08 vs. 1.40 ± 0.21, 1.80 ± 0.20 vs. 1.51 ± 0.14, 1.05 ± 0.13 vs. 0.51 ± 0.16, all *P* < 0.05; *Hla*: 2.93 ± 0.13 vs. 1.26 ± 0.08, 2.21 ± 0.11 vs. 1.82 ± 0.32, 1.36 ± 0.14 vs. 1.16 ± 0.28, 1.25 ± 0.21 vs. 0.77 ± 0.18, all *P* < 0.05, *agrA:* 5.12 ± 0.25 vs. 1.65 ± 0.22, 3.75 ± 0.36 vs. 1.42 ± 0.15, 2.11 ± 0.14 vs. 1.48 ± 0.14, 1.94 ± 0.24 vs. 1.03 ± 0.18, all *P* < 0.05).

#### Virulence proteins

**Figure 4:**
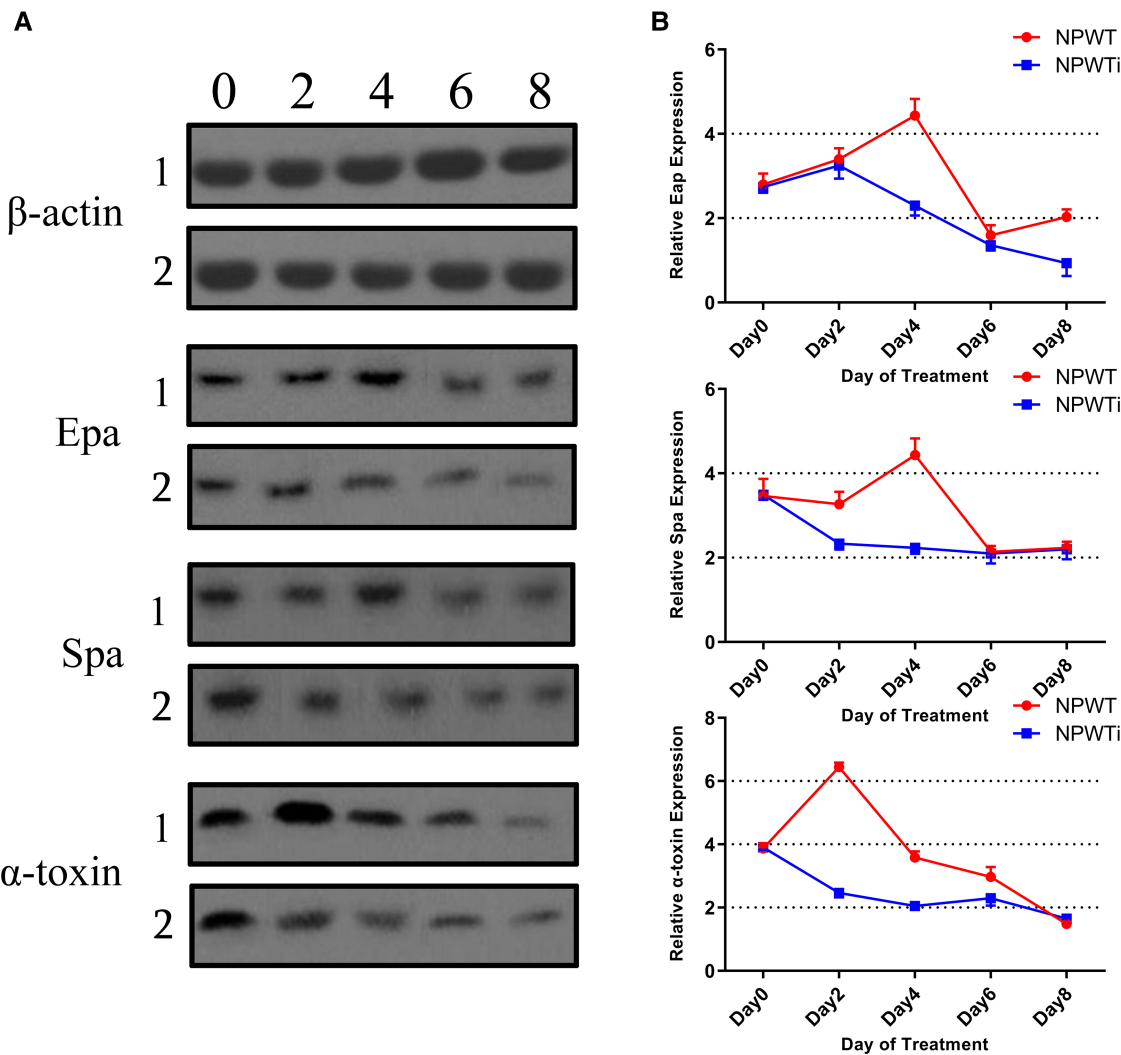
Differences in protein expression in the two treatment groups over time (fold change).

Western blot analysis was used to assess the production of Eap, Spa, and a-toxin proteins (Figure 4A). Data show no statistical difference before treatment in the two groups. The expression level of Eap protein was significantly lower in NPWTi groups than that in NPWT groups on postoperative days 4 and 8 (2.30 ± 0.10 vs. 4.43 ± 0.15 and 0.94 ± 0.15 vs. 2.03 ± 0.07, all *P* < 0.05). The expression level of Spa protein was significantly lower in NPWTi groups than that in NPWT groups on postoperative days 2 and 4 (2.33 ± 0.06 vs. 3.27 ± 0.12, 2.23 ± 0.06 vs. 4.43 ± 0.16, all *P* < 0.05). The expression level of a-toxin protein was significantly lower in NPWTi groups than that in NPWT groups on postoperative day 2 to 6 (2.47 ± 0.06 vs. 6.44 ± 0.06, 2.05 ± 0.05 vs. 3.58 ± 0.08, and 2.30 ± 0.10 vs. 2.97 ± 0.12, all *P* < 0.05), but higher in NPWTi groups than that in NPWT groups on postoperative day 8 (1.65 ± 0.05 vs. 1.48 ± 0.03, *P* < 0.05) (Figure 4B).

#### The depths of bacterial invasion

**Figure 5:**
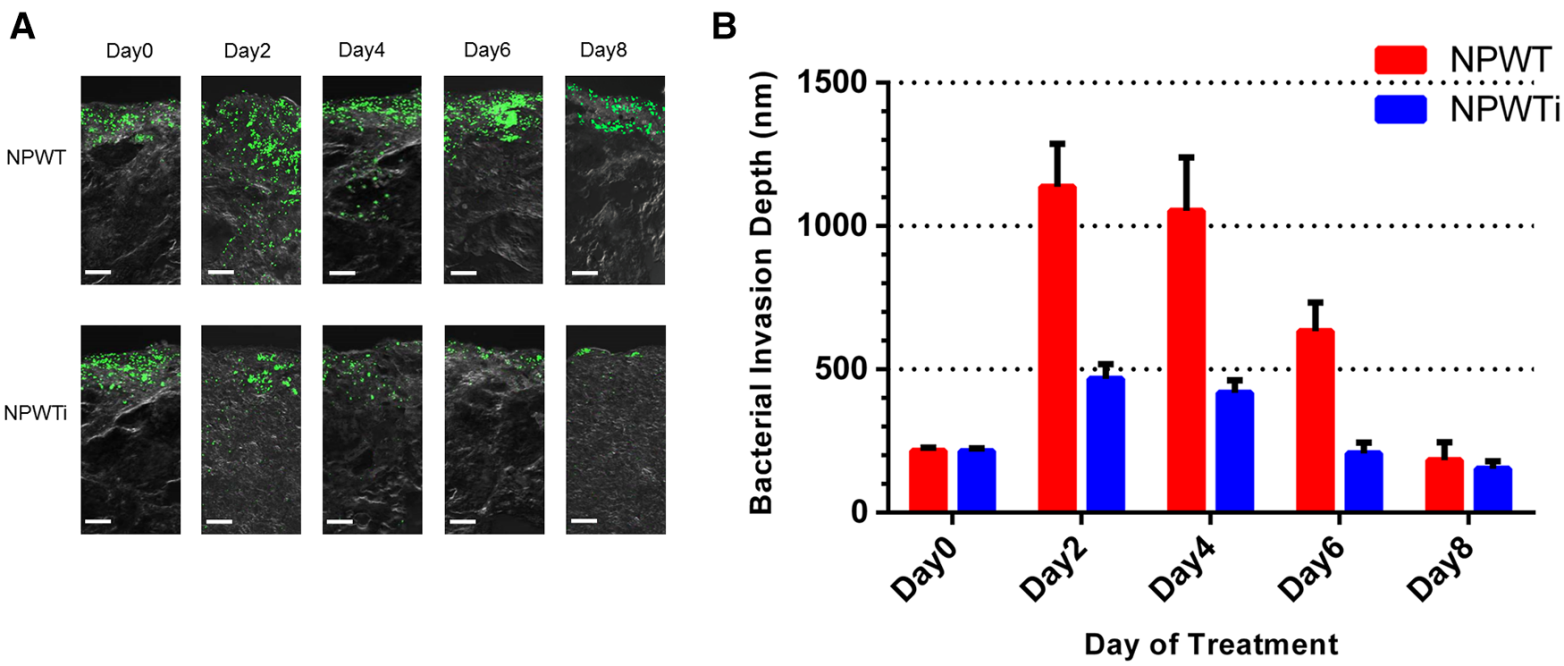
Bacterial invasion depth observed by laser scanning confocal microscopy. (**A**) This figure shows the depth of infection in NPWT and NPWTi on days 0, 2, 4, 6, and 8. (**B**) Bacterial invasion depth significantly descended in the NPWTi group compared with the NPWT group at day 2, 4, 6, and day 8 (*P < 0.001). Scale bar was 100 um.

**Figure 7:**
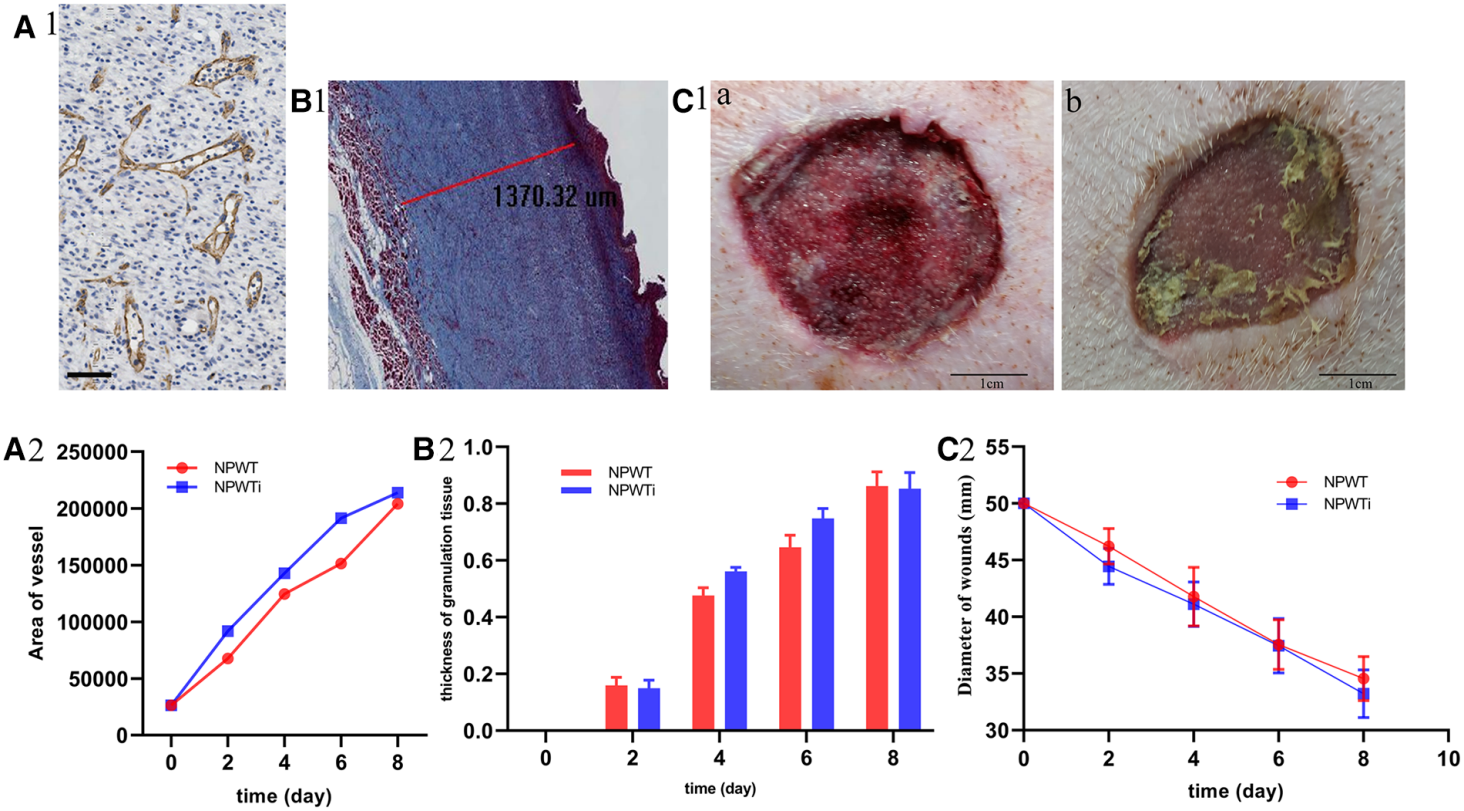
Histologic parameters and wound healing. (**A**) The difference of area of neovascularization between two groups and the scale bar was 100 um. (**B**) The difference of thickness of new granulation. (**C**) The difference of gap of wounds between two groups. Area of neovascularization and the thickness of new granulation tissue showed that it was significant larger in NPWTi group compared with NPWT on day4 and day6 (*P* < 0.05). No significant difference was observed between two groups on day 2, 4, 6, and day 8 with respect to wound gap (*P* > 0.05).

The depths of the bacterial fluorescent signal from samples before treatment and on postoperative days 2, 4, 6, and 8 were measured (Figure 5). The depth in the two groups increased on postoperative day 2 and decreased gradually from postoperative day 2 to 8. There was a significant difference on a postoperative day 2, 4, 6, and 8 between NPWT and NPWTi groups (1136.67 ± 60.28 um vs. 466.67 ± 20.82 um, 1053.33 ± 75.06 um vs. 418.33 ± 17.56 um, 633.33 ± 40.41 um vs. 206.67 ± 15.28 um, 183.33 ± 25.17 um vs. 153.33 ± 10.41 um, all *P* < 0.05) (Figure 7). The depth on a postoperative day 8 was no statistical difference compared with before treatment in the NPWT group, but a significant difference in the NPWTi group (*P* < 0.05).

### Histologic parameters and wound healing

#### Expression of *VEGF* and *bFGF*

**Figure 6:**
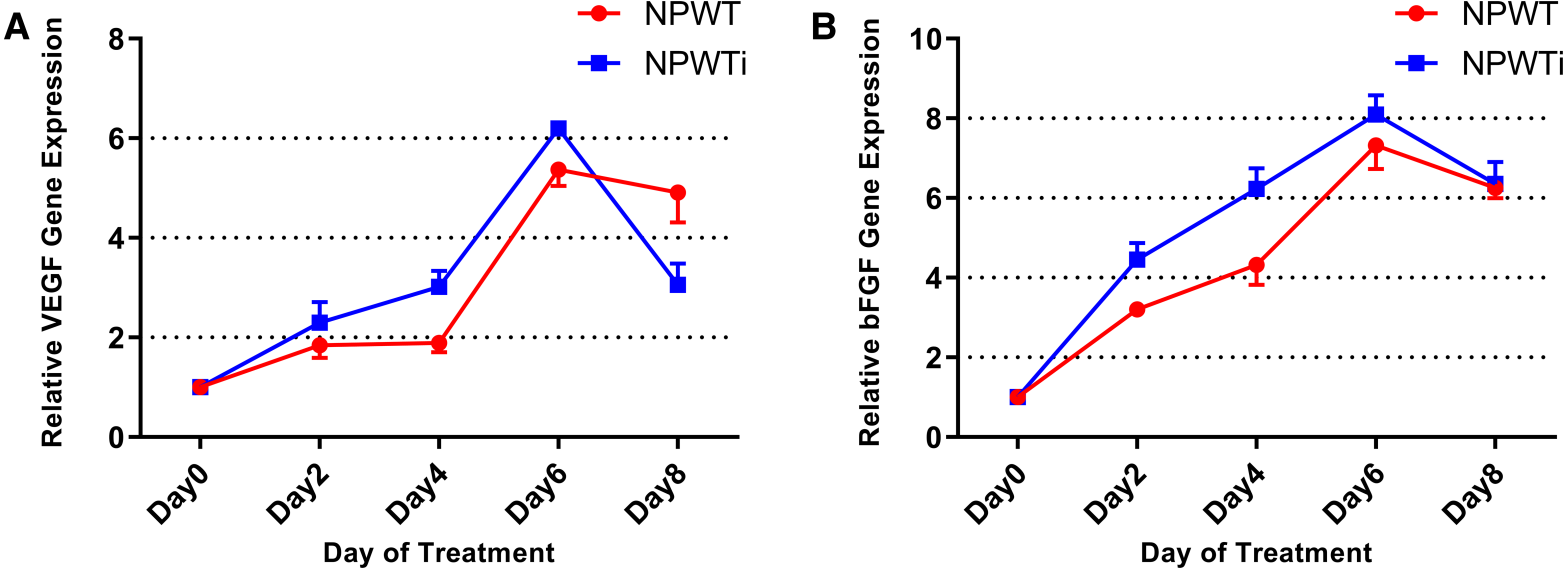
(**A**) the difference in *VEGF* expression. (**B**) The difference of *bFGF* expression. Expression levels of *VEGF* and *bFGF* on postoperative day 2, 4, and 6, were significantly higher in the NPWTi group than in that of the NPWT group (*P* < 0.05) (fold change).

In the two groups, the expression levels of *VEGF* and *bFGF* were increased gradually from postoperative day 2 to 8 (Figure 6). Expression levels of *VEGF* and *bFGF* on postoperative day 2, 4, and 6, were significantly higher in the NPWTi group than in that of the NPWT group (*P* < 0.05). However, expression levels of VEGF on postoperative day 8 were significant higher in the NPWT group. No significant difference was observed between the two groups on postoperative day 8 concerning expression levels of *bFGF* (*P* > 0.05).

#### Measurement of neovascularization, new granulation, and healing

Measurement of the area of neovascularization showed that it was significantly larger in the NPWTi group compared with NPWT on day 4 and day 6 (*P* < 0.05). No significant difference was observed between the two groups on day 8 (*P* > 0.05). Similarly, the thickness of new granulation tissue was significantly larger in the NPWTi group compared with NPWT on day 4 and day 6 (*P* < 0.05). No significant difference was observed between the two groups on day 8 (*P* > 0.05). No significant difference was observed between the two groups on days 2, 4, 6, and day 8 concerning wound gap (*P* > 0.05) (Figure 7).

## Discussion

*Staphylococcus aureus* is the most frequent opportunistic pathogen in both acute and chronic wounds. Many studies have shown a decrease in bacterial burden in response to NPWT (22, 23). Compared to NPWT, the effect of NPWTi on infected wounds is less understood. Published animal studies suggested that non-contaminated wounds may benefit from NPWTi as well (24). Up to now, the effects of NPWTi on *Staphylococcus aureus* infected wounds are still unclear.

There are many types of NPWTi, including NPWT with continuous irrigation, periodic instillation with a soak time, continuous NPWT, and regular instillation. In this experiment, we use the second type of NPWTi, which cycles between 3 discrete phases in order: instillation, soak, and NPWT. If the negative pressure is not stopped while the solution is being delivered, coverage of the wound with the instilled solution may be incomplete (25). And, Bench studies suggest that interruption of negative pressure and the introduction of a soak phase are essential for uniform coverage of the wound with the topical wound treatment solution, especially when complex wound geometries are present.

Bacteria within a wound can range from contamination, colonization, localized infection, spreading infection, and ultimately to systemic infection if not appropriately controlled. Regular instillation of solution can assist with wound cleansing, irrigation, and easier removal of infectious materials. Although the NPWTi can significantly decrease the counts of bacteria and the depths of bacterial invasion, the mechanism remains unclear. That may be what we may need to study in the next step.

Numerous studies have shown that bacterial load levels are closely related to wound healing (26–29). previous studies have reported the effects of NPWT on the bacterial burden, but the results were contradictory. A retrospective study by Weed, T. et al. showed that NPWT increased bacterial colonization significantly in patients with acute or chronic wounds (30). A randomized controlled trial (RCT) by Mouës, C. M. et al. observed that there is no significant statistical difference in the number of bacteria between NPWT and conventional therapy (31). So far, rare studies have compared the bacterial load levels between NPWT and NPWTi groups. A prospective pilot study by Goss, S. G. showed that NPWTi reduced bacterial load levels in patients with chronic wounds over 7 days, while NPWT increased bacterial load levels (32). In this study, we found that both NPWT and NPWTi can decrease the counts of bacteria, and the NPWT with instillation was superior to NPWT alone. On the postoperative day 4, the bacterial count in the NPWTi group was lower than the clinical infection standard, and on day 8, the bacterial count in both groups was lower than the clinical infection standard, but the bacterial count in the NPWTi group had reached below 10^4^ CFUs/wound. The possible reasons for the lower bacterial count in the NPWTi group may be the more efficient removal of exudates from wounds and the reduction of expression levels of *Eap*, *Spa*, and *Hla* genes.

Auto-inducing peptide (AIP), an index of bacterial density, could affect the activation of agr system. On the other hand, agr system could positively regulate the expression of a series of virulence factors, including *Eap*, *Spa*, and *Hla* genes (33). Our results showed that the expression level of *agrA* in the NPWTi group was significantly lower compared with that in the NPWT group.

Eap is probably a key factor in the pathogenesis of *S. aureus* working by mediating bacterial adhesion and endowing bacteria with the ability to evade host defense functions (34, 35). Spa protein is an important virulence factor of *S.aureus*, allowing *S.aureus* to escape host adaptive immune responses by binding to the Fc segment of immunoglobulin (36). Hla, an important pore-forming toxin (PFTs) of *S. aureus*, could promote bacterial biofilm development and macrophage dysfunction through the combined action with leukocidin AB (37). We have found that the expression level of Eap peaked on a postoperative day 4 and began to decline thereafter in both two groups. The expression level of *Spa* and *Hla* peaked on postoperative day 2 and began to decline thereafter. In general, our results showed that the expression levels of *Eap*, *Spa*, and *Hla* genes in the NPWTi group were significantly lower than that in the NPWT group. A previous study has demonstrated that changes in environmental factors, such as osmotic pressure, oxygen concentration, temperature, and other physical signal stimuli, can affect the virulence regulatory system of bacteria which plays an important role in the pathogenesis of bacteria (38). Negative pressure was a kind of physical signal stimulus and that is why it could affect the expression of these genes.

The depth of bacterial invasion in soft tissue determines the depth of infection. We have observed that the bacterial invasion depth peaked on a postoperative day 2 and began to decline thereafter, which was in accordance with the expression level of *Spa* and *Hla*. The bacterial invasion depth in the NPWTi group was significantly lower than that in the NPWT group. The possible reason is that NPWTi recirculates the perfusate through negative pressure suction, which can better reduce the incidence of blockage, reduce the load of wound secretions, and drain local secretions and necrotic tissue, so as to better take away some metabolic wastes and toxins.

*VEGF* and *bFGF* are key factors for neovascularization and wound healing. Wounds treated by NPWTi showed relatively more area of neovascularization and new granulation compared with NPWT alone. However, this superiority did not result in an effective conversion in wound healing. The potential reason is that wound healing cannot benefit from the addition of instillation. Besides, saline was used as a solution in this study, and change of solution may result in other outcomes.

There are some limitations in this study. First, only a single bacterium was used as the infection model, and wound infection in clinical practice was mostly a mixed infection of multiple bacteria. Second, wild-type *S.aureus* was used as the infection strain, which is more sensitive to antibiotics. Infection strains are often drug-resistant in clinical practice, but drug-resistant strains were not explored in this study. Third, a single solution was used in this study, and other antimicrobial solutions such as PHMB, and povidone-iodine were not investigated.

## Conclusion

This study demonstrated that NPWTi induced a better decrease in bacterial burden and virulence compared with standard NPWT on the porcine wound model. However, these advantages did not result in better wound healing.

## Data Availability

The original contributions presented in the study are included in the article/Supplementary Material, further inquiries can be directed to the corresponding author/s.
